# Intratracheal synthetic CpG oligodeoxynucleotide causes acute lung injury with systemic inflammatory response

**DOI:** 10.1186/1465-9921-10-84

**Published:** 2009-09-23

**Authors:** Sadatomo Tasaka, Hirofumi Kamata, Keisuke Miyamoto, Yasushi Nakano, Hiromi Shinoda, Yoshifumi Kimizuka, Hiroshi Fujiwara, Naoki Hasegawa, Seitaro Fujishima, Taku Miyasho, Akitoshi Ishizaka

**Affiliations:** 1 Division of Pulmonary Medicine, Keio University School of Medicine, Tokyo, Japan; 2 Department of Emergency and Critical Care Medicine, Keio University School of Medicine, Tokyo, Japan; 3 Laboratory of Veterinary Biochemistry, Rakuno Gakuen University, Ebetsu, Japan

## Abstract

Bacterial genome is characterized by frequent unmethylated cytosine-phosphate-guanine (CpG) motifs. Deleterious effects can occur when synthetic oligodeoxynucleotides (ODN) with unmethylated CpG dinucleotides (CpG-ODN) are administered in a systemic fashion. We aimed to evaluate the effect of intratracheal CpG-ODN on lung inflammation and systemic inflammatory response. C57BL/6J mice received intratracheal administration of CpG-ODN (0.01, 0.1, 1.0, 10, or 100 μM) or control ODN without CpG motif. Bronchoalveolar lavage (BAL) fluid was obtained 3 or 6 h or 1, 2, 7, or 14 days after the instillation and subjected to a differential cell count and cytokine measurement. Lung permeability was evaluated as the BAL fluid-to-plasma ratio of the concentration of human serum albumin that was injected 1 h before euthanasia. Nuclear factor (NF)-κB DNA binding activity was also evaluated in lung homogenates. Intratracheal administration of 10 μM or higher concentration of CpG-ODN induced significant inflammatory cell accumulation into the airspace. The peak accumulation of neutrophils and lymphocytes occurred 1 and 2 days after the CpG-ODN administration, respectively. Lung permeability was increased 1 day after the 10 μM CpG-ODN challenge. CpG-ODN also induced nuclear translocation of NF-κB and upregulation of various inflammatory cytokines in BAL fluid and plasma. Histopathology of the lungs and liver revealed acute lung injury and liver damage with necrosis, respectively. Control ODN without CpG motif did not induce any inflammatory change. Since intratracheal CpG-ODN induced acute lung injury as well as systemic inflammatory response, therapeutic strategies to neutralize bacterial DNA that is released after administration of bactericidal agents should be considered.

## Background

Acute respiratory distress syndrome (ARDS), which is the most severe form of acute lung injury (ALI), is a critical illness with high mortality. ALI/ARDS may occur in association with direct lung injury, including pneumonia, aspiration of gastric contents, and inhalation of noxious gas, or indirect lung injury, such as sepsis, blood transfusions and shock. Among the various predisposing factors, severe pneumonia is one of the most common causes [1]. Intratracheal administration of endotoxin (lipopolysaccharide; LPS), which is a cell wall component of gram-negative bacteria, has been extensively used as an experimental model of ALI/ARDS following severe pneumonia [2]. However, the roles of other bacterial components, such as genomic DNA, in the development of lung injury and systemic inflammatory response remain to be determined.

The bacterial genome, compared to vertebrate DNA, contains a higher frequency of unmethylated cytosine-phosphate-guanine (CpG) dinucleotides. Small oligodeoxynucleotides (ODN) with unmethylated CpG dinucleotides (CpG-ODN) are able to mimic the immunostimulatory activity of bacterial DNA since bacterial DNA and synthetic ODN share similar base sequences and bind to the same receptor system [3-5].

Toll-like receptors (TLRs) have been shown to play an essential role in the activation of innate immunity by recognizing specific patterns of microbial components [6]. Among the TLRs, TLR9 recognizes bacterial or viral DNA, although it is now known that TLR9 also recognizes unmethylated CpG-containing DNA sequences, including those of mammalian origin and synthetic ODN [4,7]. In the lung, constitutive expression of TLR9 has been detected in endothelial cells and macrophages [8,9]. It was reported that intraperitoneal or intratracheal administration of CpG-ODN causes lung inflammation [10-12]. However, little is known regarding the lung permeability changes, time course of the cytokine levels, and systemic effects of intratracheal CpG-ODN challenge.

In this study, we evaluated inflammatory cell accumulation and edema formation following intratracheal administration of CpG-ODN. Histopathology of the lungs and the liver was also analyzed. Cytokine levels in plasma and bronchoalveolar lavage (BAL) fluid were examined at various time points after CpG-ODN administration. Nuclear factor (NF)-κB DNA binding activity was evaluated using lung homogenates.

## Methods

### Materials

Synthetic CpG-ODN (5'-tccatgacgttcctgatgct-3') and control ODN without CpG motifs (5'-gcttgatgactcagccggaa-3') were purchased from HyCult Biotechnology (Uden, The Netherlands).

### Mice

Eight-week old male C57BL/6J mice were obtained from Charles River Laboratories Japan (Yokohama, Japan). Mice were given free access to water and standard rodent chow and were housed in pathogen-free cages. All animal experiments were approved by the Animal Care and Use Committee of Keio University School of Medicine.

### Murine Model of Lung Injury

Lung injury was evaluated inflammatory cell accumulation and edema formation in the lung as well as pathology. Mice (20-25 g) were anesthetized with intraperitoneal ketamine (120 mg/kg) and xylazine (12 mg/kg). Intratracheal instillation of CpG-ODN, control ODN, or vehicle (phosphate buffered saline; PBS) in a volume of 100 μL was performed via a Microsprayer (PennCentury, Philadelphia, PA). All mice were sacrificed by deep anesthesia 3 or 6 h or 1, 2, 7 or 14 days after the instillation.

### Bronchoalveolar Lavage

The mice were anesthetized using intraperitoneal pentobarbital sodium (50 mg/kg) and euthanized. The trachea was exposed, and a 20-gauge angiocatheter was inserted and secured. The lungs were lavaged with two separate 0.7-mL volumes of ice-cold PBS. The bronchoalveolar lavage (BAL) fluid was centrifuged at 400 × g for 10 min at 4°C to pellet the cell fraction, and the supernatant was stored at -80°C until the measurements of the cytokines and human serum albumin levels to calculate the permeability index. The cell pellet was resuspended in 400 μL of cold saline, and the total cell counts were determined using a hemacytometer. Differential cell counts were performed using cytocentrifuge smears stained with Diff-Quik (Sysmex, Kobe, Japan).

### Measurement of Proinflammatory Mediators in Bronchoalveolar Lavage Fluid and Plasma

BAL fluid and plasma were assayed for interleukin (IL)-1β, TNF-α, IL-6, IL-10, IL-12p40, interferon-γ (IFN-γ), monocyte chemoattractant protein-1 (MCP-1), macrophage inflammatory protein-1α (MIP-1α), MIP-1β, and keratinocyte-derived chemokine (KC) using a multiplex cytokine bead array system (Bio-Plex™; Bio-Rad, Hercules, CA) according to the manufacturer's instructions. In brief, BAL fluid supernatants or plasma were incubated with microbeads labeled with specific antibodies to one of the above-mentioned cytokines for 30 min. Samples were washed after the incubation and were then incubated with the detection antibody cocktail, each antibody specific to a single cytokine. This step was followed by another wash step, and the beads were incubated with streptavidin-phycoerythrin for 10 min, washed again, and the concentration of each cytokine was determined using the array reader. The data were analyzed using the Bio-Plex™ Manager software program.

### Lung Permeability Index

Mice were given 10 mg/kg of human serum albumin (HSA) dissolved in 100 μL of saline intravenously, 1 h before euthanasia. At the time of sacrifice, the blood was drawn from the inferior vena cava. The permeability index was defined as the ratio of the HSA concentration in the BAL fluid to that in the plasma, presented as a percentage [13]. The HSA concentration was measured using an immunoassay with a Human Albumin ELISA Quantitation Kit (Bethyl Laboratories, Montgomery, TX). The lower limit of detection was 5 ng/mL.

### Histopathology of the Lungs and the Liver

In another series of experiments, the lungs were sampled 24 h after intratracheal instillation, fixed by intratracheal instillation of 10% neutral phosphate-buffered formalin at a pressure of 22 cm H_2_O and embedded in paraffin. The liver was also fixed with 10% neutral phosphate-buffered formalin and embedded in paraffin. The tissues were cut into 3-μm sections and stained with hematoxylin-eosin for pathology.

### NF-κB (p65) DNA-binding Activity in the Lung

#### Nuclear protein extraction

After performing BAL, the left lungs were harvested and snap frozen in liquid nitrogen and then stored at -80°C until analysis. The lungs were homogenized in 2 mL of ice-cold Buffer A (10 mM HEPES, 1.5 mM MgCl_2_, 10 mM KCl, 0.5 mM DTT, 0.5 mM PMSF) with a 0.1% volume of Nonidet P-40 and a protease inhibitor cocktail (1 mg/mL leupeptin, 1 mg/mL aprotinin, 10 mg/mL soy bean trypsin inhibitor, 1 mg/mL pepstain). Following 10 min of incubation on ice, the homogenates were centrifuged at 850 × g for 10 min at 4°C. The pellets were resuspended in 2 mL of Buffer A and centrifuged at 1,200 × g for 10 min at 4°C. The crude nuclear pellets were resuspended in 40 mL of Buffer B (20 mM HEPES, 1.5 mM MgCl_2_, 0.42 M NaCl, 0.2 mM EDTA, 25% vol/vol glycerol, 0.5 mM DTT, 0.5 mM PMSF) with a protease inhibitor cocktail (as described above) and incubated for 30 min on ice. Nuclear extracts were recovered following centrifugation at 20,000 × g for 15 min at 4°C and stored at -80°C. The protein concentration of the nuclear extracts was determined using a BCA Protein Assay Kit (Pierce, Rockford, IL) with bovine serum albumin used as a standard.

#### NF-κB (p65) DNA-binding activity assay

NF-κB (p65) DNA-binding activity was examined using the TransAM™ ELISA kit (Active Motif, Carlsbad, CA) according to the manufacturer's protocol. In brief, 0.5 μg of nuclear extract was subjected to the binding of NF-κB to an immobilized consensus sequence (5'-GGGACTTTCC-3') in a 96-well plate, and the primary and secondary antibodies were added. After the colorimetric reaction, the samples were measured in a spectrophotometer at the wavelength of 450 nm. Recombinant NF-κB p65 (Active Motif) was used as a protein standard. The DNA binding specificity was assessed using wild-type or mutated oligonucleotides.

### Statistical Analysis

Data are reported as mean ± SEM. Differences among groups were determined using analysis of variance followed by post hoc analysis with the Bonferroni's test for multiple comparisons. A p value less than 0.05 was considered statistically significant.

## Results

### Accumulation of Inflammatory Cells after Intratracheal CpG-ODN Challenge

**Figure 1:**
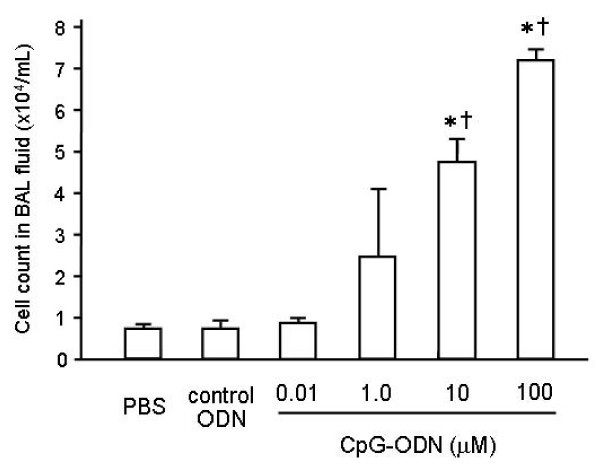
**Dose-response relationship of intratracheal CpG-ODN**. Inflammatory cell recruitment into the alveolar space was evaluated after intratracheal challenge of CpG-ODN (0.01, 0.1, 1.0, 10, or 100 μM) or vehicle (n = 6 for each group) or vehicle. Two days later, BAL fluid was obtained and subjected to a cell count. Significant inflammatory cell accumulation into the airspace was observed in the mice that received 10 μM or higher concentration of CpG-ODN. Values are the mean ± SEM; n = 5-6 for each group. *p < 0.05 compared with the PBS group. ^†^p < 0.05 compared with the control ODN group.

In the first series of experiments, mice received intratracheal instillation of CpG-ODN (0.01, 0.1, 1.0, 10, or 100 μM) or vehicle. Two days later, BAL fluid was obtained to evaluate inflammatory cell accumulation into the airspace (Figure 1). There were no significant differences in the cell counts between the PBS control animals and the mice treated with 0.01, 0.1, or 1.0 μM of CpG-ODN. The higher concentrations (10 and 100 μM) of CpG-ODN caused significant increases in the cell counts in BAL fluid compared with the PBS control animals (p < 0.05). Since we observed significant inflammatory cell accumulation into the airspace in the mice that received 10 μM or higher concentration of CpG-ODN, we used 10 μM of CpG-ODN in the following experiments.

**Figure 2:**
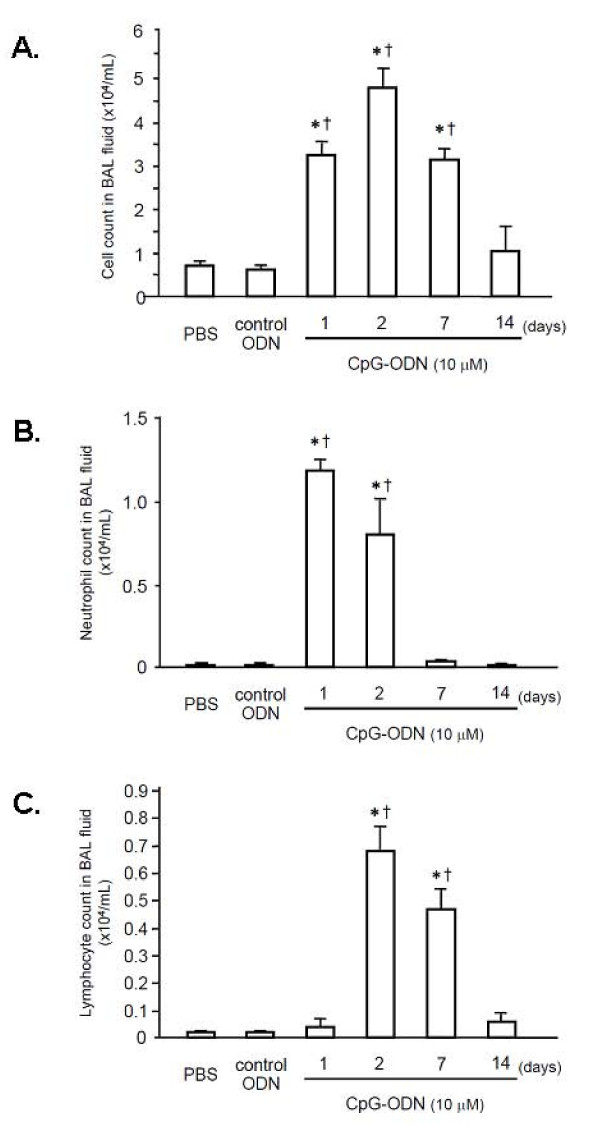
**Time course of total and differential cell counts in BAL fluid after administration of 10 μM CpG-ODN**. **(A) **Total cell counts in BAL fluid were markedly increased 24 h after CpG-ODN challenge and then decreased by 14 days after the instillation. **(B) **Neutrophil counts on days 1 and 2 were significantly greater than in the animals that received the control ODN or PBS. **(C) **Lymphocyte counts in BAL fluid were significantly increased on days 2 and 7 compared with the mice treated with the control ODN or PBS. Values are the mean ± SEM; n = 5-6 for each group. *p < 0.05 compared with the PBS group. ^†^p < 0.05 compared with the control ODN group.

In the next series, mice received intratracheal instillation of CpG-ODN (10 μM); 6 h or 1, 2, 7, or 14 days later, BAL fluid was obtained and used to perform a differential cell count. We also collected BAL fluid 24 h after the instillation of control ODN or PBS. The total and differential cell counts in BAL fluid are shown in Figure 2. CpG-ODN instillation induced a significant increase in inflammatory cell recruitment into the alveolar space, compared with the instillation of control ODN or PBS (Figure 2A). On day 1, the total cell count in BAL fluid was significantly increased compared with the control ODN and the PBS groups (p < 0.05). The total cell count was further increased on day 2 and then decreased by day 14. There was no difference in the total cell count in BAL fluid between the control ODN and the PBS groups. Neutrophil counts in BAL fluid were also markedly increased following CpG-ODN challenge (Figure 2B). On days 1 and 2, the neutrophil counts were significantly greater than in the animals treated with the control ODN or PBS (p < 0.01). The neutrophil counts then returned to the baseline by day 7. As shown in Figure 2C, lymphocyte counts in BAL fluid were significantly increased on days 2 and 7 compared with the control groups treated with the control ODN or PBS (p < 0.05). The lymphocyte counts on days 1 and 14 were not different from those in the control group. The results indicate that intratracheal administration of CpG-ODN induces neutrophil accumulation into the alveolar space within 24 h, followed by lymphocyte accumulation that lasts until 7 days after the CpG-ODN challenge.

### Changes in Lung Permeability following Intratracheal CpG-ODN Administration

**Figure 3:**
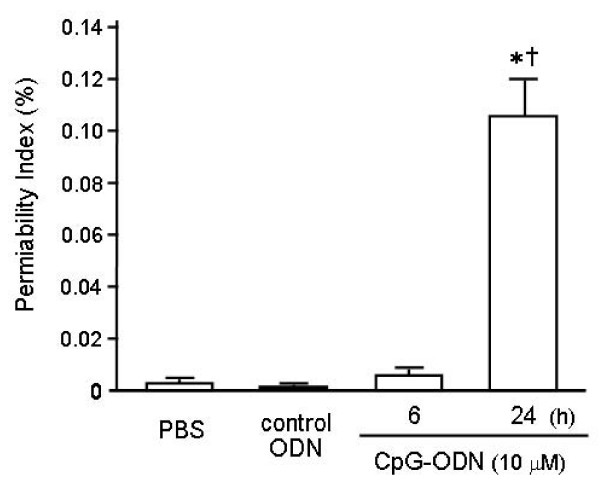
**Effect of intratracheal CpG-ODN challenge on the lung permeability**. The permeability index was calculated as the BAL fluid-to-plasma ratio of the concentration of human serum albumin injected intravenously 1 hour before sacrifice. CpG-ODN significantly increased the permeability index, which reached the peak 24 h after the instillation. Values are the mean ± SEM; n = 5-6 for each group and vehicle. *p < 0.05 compared with the PBS group. ^†^p < 0.05 compared with the control ODN group.

The permeability index was calculated as the BAL fluid-to-plasma ratio of the concentration of human albumin that was injected intravenously 1 hour before sacrifice (Figure 3). Thus, this index reflects pulmonary endothelial and alveolar septal permeability. There was no difference in the permeability index between the control animals and those sacrificed 6 h after CpG-ODN instillation (0.043 ± 0.010% vs. 0.066 ± 0.013%, respectively). The permeability index 24 h after the CpG-ODN challenge was as high as 1.043 ± 0.269%, which was significantly greater than in the other two groups (p < 0.01). These findings suggest that CpG-ODN caused not only the inflammatory cell accumulation, but also the increase in lung permeability.

### Effects of Intratracheal CpG-ODN on Cytokine Levels in BAL Fluid and Plasma

BAL fluid and plasma were collected 6 h or 1, 2, or 7 days after the intratracheal instillation, and the concentrations of IL-1β, TNF-α, IL-6, IL-10, IL-12p40, IFN-γ, MIP-1α, MIP-1β, MCP-1, and KC in the BAL fluid were measured. As shown in Table 1, CpG-ODN instillation markedly increased the levels of the pro-inflammatory cytokines and chemokines in the alveolar space within 24 h. The concentrations of most of the inflammatory mediators measured were highest 24 h after the CpG-ODN challenge, except KC, which reached its peak 6 h after the instillation. At 6 h, only KC, IL-1β, and IL-12p40 exhibited significant increases compared with the mice that received PBS or control ODN. The concentration of IL-10, an anti-inflammatory cytokine, was highest 2 days after the CpG-ODN administration. There were no significant differences in the cytokine concentrations between the mice with the control ODN instillation and those administered PBS.

**Table 1:** Cytokine levels in bronchoalveolar lavage fluid

			CpG-ODN			
			
	PBS	Control ODN	6	24	48	168 (h)
IL-1β	4.9 ± 2.1	3.4 ± 0.6	45.4 ± 3.0*†	56.7 ± 6.2*†	62.1 ± 8.0*†	12.0 ± 4.0
IL-10	3.0 ± 0.6	2.6 ± 0.4	5.1 ± 0.3	51.8 ± 12.2†	108.4 ± 23.1*†	2.6 ± 0.8
IFN-γ	2.8 ± 0.5	1.3 ± 0.3	2.0 ± 0.3	110.7 ± 38.1*†	39.9 ± 12.6	1.7 ± 0.3
TNF-α	41.2 ± 5.6	37.0 ± 1.9	75.1 ± 12.7	466.1 ± 184.1*†	153.8 ± 50.6	31.3 ± 2.5
IL-6	7.5 ± 1.7	3.5 ± 0.9	36.9 ± 8.8	150.0 ± 55.4*†	181.3 ± 69.0*†	3.2 ± 0.9
IL-12p40	15.3 ± 4.5	11.8 ± 1.6	396.9 ± 56.9*†	410.9 ± 57.8*†	453.1 ± 79.6*†	111.5 ± 45.8
KC	1.4 ± 0.3	2.0 ± 0.2	180.8 ± 38.5*†	75.0 ± 25.9	63.6 ± 16.4	2.4 ± 0.3
MCP-1	1.4 ± 0.4	1.6 ± 0.4	55.4 ± 20.7	965.9 ± 206.6*†	1295.7 ± 204.5*†	3.6 ± 2.5
MIP-1α	11.2 ± 1.4	3.6 ± 1.8	125.5 ± 50.7	321.6 ± 92.2*†	72.3 ± 15.6	4.1 ± 1.8
MIP-1β	1.6 ± 0.5	1.7 ± 0.3	17.6 ± 6.2	223.9 ± 16.0*†	158.9 ± 37.9*†	1.3 ± 0.3

All values (pg/mL) are expressed as the means ± SE (n = 5-6 in each group). *p < 0.05 was considered to be significantly different from the corresponding value of the PBS group. †p < 0.05 was considered to be significantly different from the corresponding value of the control ODN group.

In plasma, CpG-ODN also induced upregulation of IL-1β, IL-6, IL-10, IL-12p40, KC, MCP-1, and MIP-1β, compared with the animals receiving control ODN administration (Table 2). The plasma level of IL-12p40 was significantly elevated as early as 6 h after the CpG-ODN challenge. The levels of other pro-inflammatory mediators in plasma reached their peaks at 24 h, although the IL-10 level was highest 48 h after the instillation, as was the case in BAL fluid. Plasma cytokine levels returned to the baselines by day 7. There were no significant differences in the plasma cytokine concentrations at any time point between the mice with the control ODN instillation and those administered PBS.

**Table 2:** Cytokine levels in plasma

			CpG-ODN			
			
	PBS	Control ODN	6	24	48	168 (h)
IL-1β	34.7 ± 6.3	28.8 ± 6.6	47.6 ± 14.9	56.6 ± 5.6*†	42.4 ± 6.0*†	25.0 ± 4.4
IL-10	39.0 ± 0.6	33.5 ± 4.1	35.6 ± 13.0	95.2 ± 17.0	122.3 ± 18.5*†	46.1 ± 16.8
IFN-γ	2.3 ± 1.0	10.6 ± 4.3	1.7 ± 0.6	12.5 ± 4.8	4.2 ± 1.8	8.3 ± 7.5
TNF-α	759.0 ± 96.8	664.2 ± 78.0	550.3 ± 64.8	777.7 ± 83.0	690.8 ± 135.8	633.2 ± 74.0
IL-6	29.3 ± 18.8	14.1 ± 2.2	39.0 ± 7.0	124.3 ± 17.6*†	46.2 ± 9.3	12.7 ± 6.2
IL-12p40	126.0 ± 4.8	116.4 ± 10.9	314.5 ± 65.8*†	303.9 ± 20.9*†	235.8 ± 30.2	120.2 ± 12.4
KC	31.9 ± 4.9	32.0 ± 4.1	53.1 ± 13.4	92.7 ± 12.4*†	47.8 ± 9.8	23.2 ± 3.7
MCP-1	16.0 ± 5.7	4.6 ± 3.9	48.2 ± 27.0	137.1 ± 41.5*†	25.9 ± 9.5	13.6 ± 3.5
MIP-1α	31.5 ± 4.4	29.2 ± 3.4	25.5 ± 7.6	65.0 ± 8.9	42.0 ± 7.2	21.3 ± 11.2
MIP-1β	8.5 ± 2.5	6.3 ± 3.4	8.4 ± 3.4	23.6 ± 4.4†	10.7 ± 2.8	2.7 ± 1.3

All values (pg/mL) are expressed as the means ± SE (n = 5-6 in each group). *p < 0.05 was considered to be significantly different from the corresponding value of the PBS group. † p < 0.05 was considered to be significantly different from the corresponding value of the control ODN group.

### Histopathological Changes in the Lungs and Liver Induced by CpG-ODN

**Figure 4:**
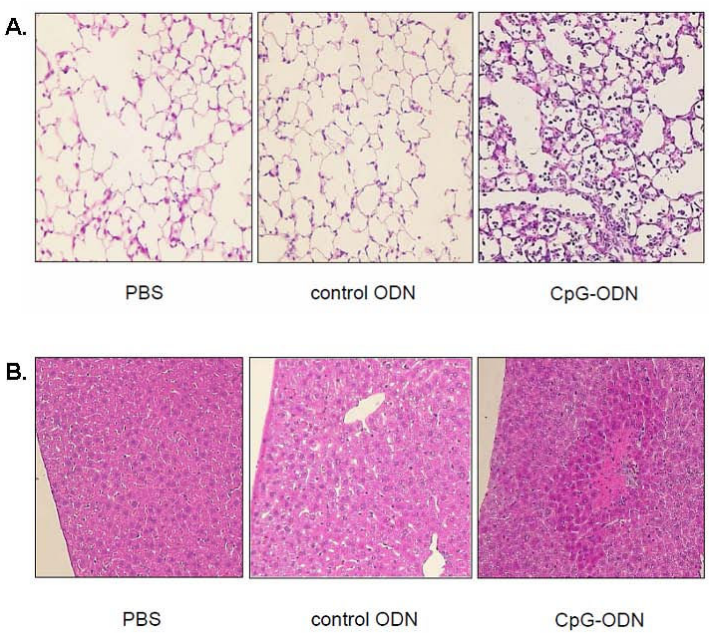
**Representative histopathological findings of the lungs and the liver after CpG-ODN challenge**. **(A) **CpG-ODN challenge caused numerous neutrophil accumulation in the alveolar space with thickening of the alveolar septa and areas of hemorrhage. **(B) **Intratracheal CpG-ODN induced marked liver damage, including disruption of normal architecture, necrosis, neutrophil infiltration, and hemorrhage. Original magnification × 100.

Representative microscopic findings of the H-E-stained lung specimens are shown in Figure 4A. In the mice treated with PBS or control ODN, no obvious neutrophil recruitment was observed at 24 h after the intratracheal instillation. The CpG-ODN challenge caused marked neutrophil accumulation in the alveolar space with thickening of the alveolar septa and areas of hemorrhage, which is suggestive of acute lung injury.

Since we observed elevated levels of inflammatory mediators in plasma, the liver was also subjected to histopathology. As shown in Figure 4B, the livers from the mice, which received CpG-ODN intratracheally, revealed marked damage, including disruption of normal architecture, necrosis, neutrophil infiltration, and hemorrhage. The mice treated with PBS or control ODN showed normal liver lobular architecture and hepatocytes.

### Effect of CpG-ODN on NF-κB DNA-binding Activity in the Lung

**Figure 5:**
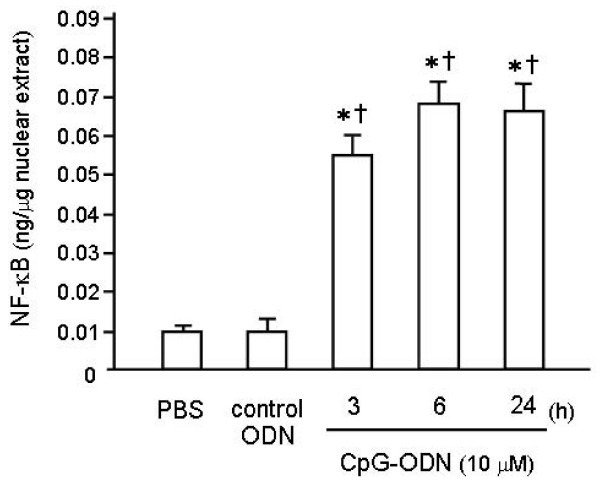
**Effect of intratracheal CpG-ODN challenge on NF-κB DNA-binding activity in the lung**. To evaluate the effect of CpG-ODN on the NF-κB signaling pathway, nuclear extracts of lung homogenates were analyzed using the TransAM™ ELISA kit. CpG-ODN significantly increased NF-κB DNA-binding activity, which reached the peak 24 h after the instillation. Values are the mean ± SEM; n = 5-6 for each group. *p < 0.05 compared with the PBS group. ^†^p < 0.05 compared with the control ODN group.

To evaluate the effect of CpG-ODN on the upregulation of the NF-κB signaling pathway in the lung, nuclear extracts of lung homogenates were analyzed using the TransAM™ ELISA kit. Because the most frequently activated form of NF-κB in TLR signaling is a heterodimer composed of Rel A(p65)-p50 [14] and p50 lacks the transcription activation domain, we used p65 as a marker of NF-κB activation. As shown in Figure 5, CpG-ODN challenge induced high levels of NF-κB DNA-binding activity within 6 h, which had been weakened to the baseline by day 2. Additionally, the binding was specific, since the wild-type consensus oligonucleotide prevented NF-κB binding to the probe immobilized on the plate; conversely, the mutated oligonucleotide had no effects on NF-κB binding (data not shown).

## Discussion

In this study, we demonstrated that intratracheal administration of CpG-ODN induces inflammatory cell accumulation and permeability edema, as well as upregulation of inflammatory mediators in the alveolar space. In addition, CpG-ODN challenge caused a systemic inflammatory response characterized by hypercytokinemia and liver damage. In contrast, control ODN without CpG motif did not exert such pathological effects. These results suggest that microbial DNA, which can be released during antibiotic treatment, might contribute to not only the development of ALI/ARDS but also the systemic inflammatory response following pneumonia. To the best of our knowledge, this is the first report on the effect of intratracheal administration of synthetic CpG-ODN on local and systemic inflammatory responses.

Previous studies indicated that CpG-ODN enhances antimicrobial immunity when it is administered systemically or inhaled [15-17]. For example, Deng and colleagues demonstrated that pretreatment with intratracheal CpG-ODN improved survival after intratracheal inoculation of *Klebsiella *[15]. On the other hand, it has been reported that deleterious effects can occur when bacterial DNA or CpG-ODN is administered in a systemic fashion [12,18]. Although compartmentalized delivery of CpG-ODN has been reported to be well tolerated and protective against respiratory pathogens, our results showed harmful effects of intratracheal CpG-ODN. This discrepancy might be due to the relatively larger amount (1.0 nmol) of CpG-ODN used in the present study. Taken together, CpG-ODN, which enhances host defense, may cause tissue injury when administered in excess.

Neutrophils have been recognized as important contributors to the pathogenesis of ALI/ARDS [1,19]. In this study, intratracheal administration of CpG-ODN induced marked neutrophil influx into the alveolar space. In addition, the liver pathology showed neutrophil infiltration with hemorrhage and necrosis at 24 h, suggesting that upregulation of inflammatory mediators could have occurred at an earlier time point. We observed that plasma levels of KC and IL-12p40 were elevated 6 h after the CpG-ODN challenge, whereas the levels of other mediators did not differ from those in the mice treated with the control ODN or PBS at the time point. In mice, KC or CXCL8 is one of the most important chemokines for neutrophil recruitment [20]. Since we observed markedly elevated KC levels in BAL fluid as early as 6 h after the instillation of CpG-ODN, KC might be a key mediator that promoted neutrophil accumulation into the lung. In addition, plasma KC levels were elevated at 24 h, suggesting that KC might contribute to further neutrophil recruitment into the lungs and liver. Stefanovic and Stefanovic reported that KC overexpression causes massive liver necrosis within 2 days and KC induces immediate expression of proinflammatory genes [21]. We concluded that KC might play a central role in the pulmonary and systemic inflammation following CpG-ODN challenge.

IL-12, a crucial cytokine for DC-mediated induction of Th1 cell differentiation, is also known as a T cell stimulating factor, which stimulates the production of IFN-γ and TNF-α from T and natural killer cells. It was previously reported that CpG-DNA stimulation resulted in prolonged NF-κB activity at the IL-12p40 promoter and IL-12p40 production by hepatic DCs [22,23]. In addition, Tanaka and colleagues reported that anti-IL-12 mAb blocked *Propionibacterium acnes *and LPS-induced liver injury and concluded that IL-12 may be an essential cytokine in the course of T cell-dependent liver injury [24]. In the present study, IL-12p40 was the only mediator that was elevated in plasma as early as 6 h. We believe that CpG-ODN stimulation might induce the production of IL-12, which could play a key role in the development of the liver injury.

We previously reported that LPS-induced pulmonary responses can be augmented by mononuclear cell phagocytosis in the reticuloendothelial system (RES) [25]. The activated RES might contribute to the further upregulation of inflammatory mediators. It remains to be determined whether, at the later time points, plasma inflammatory mediators are released from the alveolar space or RES in the liver or spleen.

There are two possible mechanisms for the remote liver damage we observed. One is that the liver damage was caused by inflammatory mediators that leaked or were released from the alveolar space. The other is that the injury was caused directly by CpG-ODN that entered the blood stream from the lungs. Slotta and coworkers reported that intraperitoneal injection of CpG-ODN caused liver injury on day 1, which was characterized by sinusoidal leukostasis, deterioration of the microcirculation, and hepatocyte apoptosis [26]. Considering the molecular size of CpG-ODN (2.6 kDa), we speculated that CpG-ODN could travel through the epithelial and endothelial barriers of the lung into the blood stream.

Severe pneumonia is known to be one of the major risk factors for the development of ALI/ARDS [1]. It was also reported that ALI/ARDS can develop even though appropriate antibiotics are administered to treat the pneumonia [27]. When bacteria are killed by antibiotics, the bacterial cell component or its fragments are released into the surrounding environment, such as blood or alveolar epithelial lining fluid. It is widely known that cell wall components, such as LPS and peptidoglycan, can cause acute lung injury via the TLR4 and TLR 2 signaling pathways, respectively [6]. The results of our study indicate that microbial DNA could also contribute to the pathogenesis of tissue injury. The pathogen-associated molecular pattern of unmethylated bacterial DNA is recognized by TLR9, and its immunomodulatory effects can be mimicked by ODNs containing unmethylated CpG motifs [4,5]. Upon recognition of CpG-rich sequences in the endosome, TLR9, expressed by B cells, macrophages, and dendritic cells (DCs), initiates a conserved TLR family signaling cascade that begins with the recruitment of the adaptor protein MyD88, resulting in NF-κB translocation to the nucleus [28]. CpG-induced NF-κB activation initiates the up-regulation of costimulatory molecules and the secretion of proinflammatory cytokines, such as TNF-α and IL-6. Since we observed significant NF-κB activation as well as up-regulation of inflammatory cytokines following CpG administration, this pathway could be responsible for the development of pulmonary and systemic inflammation.

It may be difficult to extrapolate the present data to clinical settings, partly because the number or frequency of CpG motifs in a genome is varied between the types of organisms. In addition, the local concentration of CpG-ODN is determined by not only the bacterial burden but also the amount of the fluid in the airspace. For example, a pneumonic patient with dehydration, who has lesser amount of the exudate and shows modest infiltration on the chest roentgenogram, may have concentrated CpG-ODN in the airspace.

The major limitation of this study is that it still remains unclear whether or not various inflammatory events after the CpG-ODN challenge are truly TLR9-dependent. Several reports have described TLR9-independent recognition of bacterial DNA [29,30]. For instance, transfection with bacterial double stranded DNA activates macrophages and DCs to produce TNF in a TLR9-independent manner [29]. In addition, DAI-1, previously named DLM-1 or Z-DNA binding protein 1, has been identified as a cytoplasmic receptor that senses and is activated by DNA, leading to type I IFN gene induction [31]. The mechanisms of CpG-ODN-induced pulmonary and systemic inflammatory processes should be the subject of future investigation.

## Conclusion

Intratracheal administration of CpG-ODN induced inflammatory cell accumulation and permeability edema of the lung as well as cytokinemia and inflammatory change in the liver. It is concluded that microbial DNA could cause lung injury with systemic inflammation. Among various microbe-derived products, we may have to consider not only cell wall components such as LPS, but also gene fragments as a key material and therapeutic target to prevent ALI/ARDS following pneumonia.

## Conflict of interests

The authors declare that they have no competing interests.

## Authors' contributions

ST designed the study and drafted the manuscript. HK carried out most in vivo experiments and performed the statistical analyses. KM, YN, HS, YK, HF, NH and SF helped the experiments. TM was engaged on cytokine measurement using suspension assay. AI supervised the study. All authors read and approved the manuscript.
